# Evaluation of antioxidant and anticancer activity of crude extract and different fractions of *Chlorella vulgaris* axenic culture grown under various concentrations of copper ions

**DOI:** 10.1186/s12906-020-03194-x

**Published:** 2021-02-05

**Authors:** Eman A. El-fayoumy, Sanaa M. M. Shanab, Hanan S. Gaballa, Mohamed A. Tantawy, Emad A. Shalaby

**Affiliations:** 1 Department of Botany and Microbiology, Faculty of Science, Cairo University, Giza, 12613 Egypt; 2 Department of Biochemistry, Faculty of Agriculture, Cairo University, Giza, 12613 Egypt; 3 Department of Hormones. Medical Research Division, National Research Centre, Dokkie, Egypt

**Keywords:** *Chlorella vulgaris*, Antioxidant and anticancer activity, Growth rate, Active ingredients

## Abstract

**Background:**

*Chlorella vulgaris* is a microalga potentially used for pharmaceutical, animal feed, food supplement, aquaculture and cosmetics. The current study aims to study the antioxidant and prooxidant effect of *Chlorella vulgaris* cultivated under various conc. of copper ions.

**Methods:**

The axenic green microalgal culture of *Chlorella vulgaris* was subjected to copper stress conditions (0.00, 0.079, 0.158, 0.316 and 0.632 mg/L). The growth rate was measured at OD_680_ nm and by dry weight (DW). Moreover, the Antioxidant activity against DPPH and ABTS radical, pigments and phytochemical compounds of the crude extracts (methylene chloride: Methanol, 1:1) were evaluated. The promising Cu crude extract (0.316 mg/L) further fractionated into twenty-one fractions by silica gel column chromatography using hexane, chloroform and ethyl acetate as a mobile phase.

**Results:**

The obtained results reported that nine out of these fractions exhibited more than 50% antioxidant activity and anticancer activity against Hela cancer cell lines. Based on IC_50_, fraction No. 7 was found to be the most effective fraction possessing a significant increase in both antioxidant and anticancer potency. Separation of active compound (s) in fraction No 7 was performed using precoated silica gel plates (TLC F_254_) with ethyl acetate: hexane (9:1 v/v) as mobile phase. Confirmation of active compound separation was achieved by two-dimensional TLC and visualization of the separated compound by UV lamp. The complete identification of the separated active compound was performed by UV- Vis- spectrophotometric absorption, IR, MS, H^1^-NMRT C^13^-NMR. The isolated compound ((2E,7R,11R)-3,7,11,15-Tetramethyl-2-hexadecenol) have high antioxidant activity with IC_50_ (10.59 μg/ml) against DPPH radical assay and comparable to the capacities of the positive controls, Butylated hydroxy toluene [BHT] (IC_50_ 11.2 μg/ml) and Vitamin C (IC_50_ 12.9 μg/ml). Furthermore, pure isolated compound exhibited a potent anticancer activity against Hela cell line with IC_50_ (4.38 μg/ml) compared to Doxorubicin (DOX) as synthetic drug (13.3 μg/ml). In addition, the interaction of the pure compound with Hela cancer cell line and gene expression were evaluated.

**Conclusions:**

The authors recommend cultivation of *Chlorella vulgaris* in large scale under various stress conditions for use the crude extracts and semi purified fractions for making a pharmaco-economic value in Egypt and other countries.

## Background

An interest in the production of bioactive compounds from natural sources has lately emerged, driven by a growing number of scientific studies that demonstrate their beneficial effects on health [1–4]. In this regard, different bioactive compounds from natural sources can be used for the treatment of various human diseases such as anticancer, antibacterial, antiviral, antiinflammation, antioxidant ---etc. as reported by [5–11].

Several studies have been executed to investigate products of microalgae metabolism not only to understand its nature but also to explore substances with possible applications to humans in various fields of interest [12]. *Chlorella Sp* is a microalga potentially used for pharmaceutical, animal feed, food supplement, aquaculture and cosmetics [13–17]. It can be cultured and harvested easily, having short generation times enabling an environmentally friendly approach for drug discovery overcoming problems that associated with the over-vitalization of marine resources and use of distractive collection practices [18].

The algal extracts analysis has recently been studied for their significant efficiency in the hindrance of different diseases. However, only a few studies have been performed regarding the phytochemical, antioxidant, and pharmacological activities of algae [11, 17, 19, 20]. Cancer has become one of the world’s leading adverse health effects with significant social and economic implications. Currently, cancer incidence rates account for one in seven deaths in the world, with far more deaths than tuberculosis, AIDS and malaria combined [17, 21]. Radiotherapy and chemotherapy for cancer therapeutics have many side effects such as the efficacy and non-specificity, chemo-resistance, and the responses of patients’ immune systems. Natural compounds are an extremely valuable resource of biologically active moieties which play a major role in the quest for new drugs by acting as lead molecules for the discovery of new drug candidates [22]. At present, the percentage of drugs derived from natural sources with anticancer potential, is quite high corresponding to approximately 60% [23].

Microalgal compounds have been associated to immune response stimulation [24], as well as cytotoxic response against numerous cancer cell lines [8, 14].

The nutrimental factors are considered as manipulation of culture media composition (carbon source, nitrogen, phosphorus and iron deficiency), while physical is described as manipulation in operation conditions and external factors that affect the microalgae growth (high light intensities, temperature, pH, salinity and electromagnetic fields) [25, 26].

Recently, several studies on the impact of multiple stresses including heavy metal exposure to microalgae growth was performed [27]. Algae often can minimize free radical damage inducing an antioxidant defensive system [28]. Both algal extracts and extracellular products have supported their antitumor, antioxidant, antimicrobial and antiviral activities [29]. Cu is considered an element which is vital growth of photosynthetic species. In high levels it become phytotoxic to cells, those significantly inhibit growth and lead to cell death [30]. Cu stress induced decrease in both pigment contents and growth rate in microalgae [31] and increasing the ROS generation through Cu interference in Fenton’s reaction [28]. It rapidly attacks biomolecules (DNA, protein and lipid) leading to metabolic dysfunction and cell death [32].

The current study aims to evaluate the potential use of *Chlorella vulgaris* cultivated under Cu stress conditions as antioxidant in addition to its prooxidant effect with separation and identification of active ingredients. Furthermore, the expression of pro-apoptotic and anti-apoptotic genes were evaluated.

## Methods

### Chemicals and reagents

All organic solvents, silica gel, TLC (F_254_) were purchased from E. Merck Co. (Darmstadt, Germany). DPPH, ABTS^.+^ were purchased from Sigma-Aldrich (St. Louis, MO, USA).

### Anticancer activity

#### Cell line cultures

Human cervical cancer (Hela) was obtained and propagated in the center for genetic engineering, Faculty of Medicine, Al Azhar University, Egypt.

### Antioxidant kits

#### Lipid peroxidation (MDA) colorimetric/ Fluorometric assay kit

Kit includes MDA lysis buffer, phosphotungestic acid solution, TBA, BHT and MDA standard.

### Gene expression

1. RNA extraction

RNA was extracted from treated and untreated Hela cells after 48 h using Gene JET RNA purification kit according to the manufacturer’s protocol.

2 Reverse transcription
  Reverse transcription reaction mixture “QuantiTect Reverse Transcription kit”
3 Verification of cDNA synthesis: GAPDH specific control primers (designed to be complementary to human GAPDH genes) were used to verify the synthesis of cDNA produced from the extracted RNA.
4 Quantitative real-time Polymerase Chain Reaction (RT-PCR)

Evaluation of the expression of pro-apoptotic genes (p^53^, Caspase 3 and Bax) and anti-apoptotic genes (Bcl-2) using RT-PCR. Quantitative real-time PCR was performed on a Rotor-Gene Q cycler was carried out using the newly synthesized cDNA as templates for PCR and using QuantiTect SYBR Green PCR kits and forward and reverse primers for each gene.

22 The nucleic acid sequences of the primers were as follows

**Table:** 

Gene	Forward primer	Reverse primer
CASP-3-	5′-TTC ATT ATT CAG GCC TGC CGA GG-	5′-TTC TGA CAG GCC ATG TCA TCC TCA-3′
Bcl-2	5′-CCTGTG GAT GAC TGA GTA CC-3’	5’-GAGACA GCC AGG AGA AAT CA-3′
Bax	5ˈ-GTTTCATCCAGGATCGAG CAG-3’	5′-CATCTTCTTCCAGATGGT GA-3’
P^53^	5-‘CCCCTCCTGGCCCCTGTCATCTTC-3’	5′-GCAGCGCCTCACAACCTCCGTCAT-3’

#### Algal cultivation

The green microalga *Chlorella vulgaris* used in this study was kindly isolated and identified by Dr. Sanaa Shanab, Professor of Phycology in the Department of Botany and Microbiology, Faculty of Science, Cairo University, according to [33, 34], then molecularly identified by sigma company (6th October, Egypt). The alga was cultivated on BG11 medium [35] and incubated at controlled culture conditions of temperature (25±2 °C) light intensity (40 μmol m^− 2^ s^− 1^), light duration (16-8 L/D cycles) with continuous aeration (60bubbles/min.).

#### Preparation of axenic culture (free from bacteria)

Mixture of antibiotics; penicillium G, dihydrostreptomycin sulfate and gentamycin sulfate at different concentrations according to the method described by [36]. The axenic culture of the alga was used in all experimental work.

#### Modification of the chemical composition of the culture medium

Studying the effect of copper concentrations provided by the culture medium (BG11), an increase or decrease of certain element concentration was performed (as single element stress). copper concentrations were used as the following: Zero, 0.158, 0.316 and 0.632 mg /L.

#### Extraction of *Chlorella vulgaris*

The dried algal biomass was extracted three times with organic solvent mixture of methanol and methylene chloride (1:1) for 40 min followed by centrifugation, filtration and evaporation of solvents using rotary evaporator at 40–50 °C. The obtained crude extract was expressed as percentage of the dried biomass weight used (mg extract/g dry biomass weight).

**Table:** 

Solvent	Fractions No.																				
	1	2	3	4	5	6	7	8	9	10	11	12	13	14	15	16	17	18	19	20	21
Hexane	100	90	80	70	60	50	40	30	20	10	0	0	0	0	0	0	0	0	0	0	0
Chloroform	0	10	20	30	40	50	60	70	80	90	100	100	90	80	70	60	50	40	30	20	10
Ethyl acetate	0	0	0	0	0	0	0	0	0	0	0	0	10	20	30	40	50	60	70	80	90

#### Fractionation of *C.vulgaris* (0.316 g/L cu)

The chromatographic column (40 cm length, 2.5 cm diameter) was packed with 150 g silica gel (60–120 mesh for column chromatography) using Hexane as solvent. 3 g of Methylene chloride: Methanol (1:1) crude extract of *C.vulgaris* cultivated under 0.316 mg/L Cu (as promising crud extract) were grounded very well with some of silica gel powder and then placed on the top of the packed column*.* The column was then sequentially eluted with 100% Hexane and increased the polarity with chloroform followed by ethyl acetate, the polarity increased by 10% between each mobile phase mixtures (total 21 fractions were obtained) as the following:

### Biological activities of extract and fractions

#### Antioxidant activity

**-**DPPH radical scavenging activity

The scavenging effect of algal extract and fractions were determined by the method of [37].

-ABTS radical cation scavenging assay.

This assay was based on the ability of different substances to scavenge (2, 2′- azino-bis ethylbenzthiazoline-6-sulfonic acid (ABTS^. +^) according to method described by [38].

#### Anticancer activity

Cytotoxic effects of extract and fractions were tested against Hela cell lines (by MTT assay [39, 40] using 96-well plate in triplicates and fractions were dissolved in DMSO were tested against Hela cell lines, Data were calculated as percentage of cell viability.

#### Gas chromatography–mass spectrometry (GC/MS) analysis

The chemical composition of promising algal extracts was performed using Trace GC1300-TSQ mass spectrometer (Thermo Scientific, Austin, TX, USA) with a direct capillary column TG–5MS (30 m × 0.25 mm × 0.25 μm film thickness). The components were identified by comparison of their retention times and mass spectra with those of WILEY 09 and NIST 11 mass spectral database.

#### Prooxidant effect of pure *Chlorella vulgaris* compound

- Determination of Reactive oxygen species

Treated Hela cell line incubated with pure compound for 48 h was rinsed, homogenized in 20 ml of 1X PBS and stored overnight at ≤ − 20 °C after two freeze-thaw cycles were performed to break the cell membranes, the homogenates were centrifuged for 5 min at 5000 rpm. Remove the supernatant and assay immediately or store at ≤ − 20 °C for determination of reactive oxygen species. Creation of a standard curve by plotting the mean absorbance for each standard on the x-axis against the concentration on the y-axis the data may be linearized by plotting the log of the ROS concentrations versus the log of the OD and the best fit line can be determined by regression analysis [41].

b. Lipid Peroxidation (MDA)

Hela cell line incubated with pure compound for 48 h, it can be homogenized on ice in 300 μl of MDA Lysis Buffer (with 3 μl BHT (100X), then centrifuged (13,000 rpm. For 10 min.) to remove insoluble material. Alternatively, protein can be precipitated by homogenizing 10 mg sample in 150 μl ddH_2_O + 3 μl BHT and adding 1 vol of 2 N perchloric acid, vortexing, and centrifuging to remove precipitated protein. Place 200 μl of the supernatant from each sample into a microcentrifuge tube. Add 600 μl of TBA reagent into each vial containing standards and sample. Incubate at 95 °C for 60 min. Cool to room temperature in an ice bath for 10 min. Pipette 200 μl (from each 800 μl reaction mixture) into a 96-well microplate for analysis occasionally, samples will exhibit a turbidity which can be eliminated by filtering through a 0.2 μm filter. For colorimetric analysis, Read the absorbance at 532 nm.

iii. Gene expression

RNA was extracted from each sample treated cell lines using nuclease free-water [42] and stored at − 70 °C. Concentration and purity of the extracted RNA were determined according to manufacturer’s protocol, where RNA was diluted with distilled water and the optical density was measured spectrophotometrically at 260 and 280 nm and the expected absorbance range of pure extracted RNA should be within 1.7 to 2.1 according to [43] For each sample, extracted RNA (1 μg), random hexamer primer (1 μl) and DEPC-treated water (to12 μl) were mixed, centrifuged briefly and incubated at 65 °C for 5 min. Samples were placed on ice and the following components were added to each sample in the indicated order as recorded in “QuantiTect Reverse Transcription kit”. The real-time PCR mixture was pre-denaturized at 94 °C for 3 min. 35 cycles with each cycle consisting of denaturation at 94 °C for 30 s, followed by extension at 72 °C for 45 s. Reactions were terminated by heating at 72 °C for 5 min. Non-reverse transcribed RNAs were included to confirm the absence of genomic DNA. A negative control without adding template was also included to assess for reagent contamination RT-PCR product as 10 μl was loaded on 1.5% agarose gel. Band detection was achieved at 100 V for 20–30 min, using UV transilluminator and photographed after staining using Ethidium Bromide, PCR product should be visible at 496 bp [44].

### Thin layer chromatography

The separation of active compound from the promising fraction of *C.vulgaris* was performed using precoated silica gel plates (TLC F_254_) using Hexane: ethyl acetate (1:9 v/v) as mobile phase, the separated spot was scratched. Two dimensions TLC was used for confirmation the purity of this spot. Complete identification of the compound was performed using Mass Spectrum (MS), Infrared (IR) as well as Nuclear Magnetic Resonance Spectroscopy (^1^H-NMR and ^13^C NMR) as the following:

### Mass spectrum

The bioactive compound separated from the promising fraction of the algal biomass crude extract was analyzed by Mass spectrum (MS) at Micro analytical center/ Faculty of Science Cairo University. The Mass spectrometer was scanned over the range of 40–500 nm/z with an ionizing voltage of 70 ev and identification was based on standard mass library of National Institute of Standard and Technology (NIST Version 2.0).

### Infrared spectrum

Using Perkin Elmer 1430 infrared spectrophotometer (400–4000 nm), the molecular structure of the separated bioactive compound was partially identified through the presence of chemically active groups (functional groups).

### Ultraviolet - visible spectroscopy

Ultraviolet - visible spectrophotometry related to the spectroscopy of photons in the UV- visible region. The separated compound was scanned from 400 to 500 nm range at 10 nm interval in the ultraviolet - visible spectrometer against solvent blank. The spectrum was recorded.

### H^1^-NMR and C^13^NMR nuclear magnetic resonance

H^1^-NMR spectrum of the pure compound was performed by dissolving it in dimethyl sulfoxide (DMSO). The different protons of functional groups of the compound could be identified using H^1^-NMR (Varian Gemini 200 MHZ). C^13^NMR heteronuclear single quantum correction was carried out using the Brucker’s standard pulse library.

### Statistical analysis

All the data are expressed as mean ± standard deviation of three determinations. Statistical comparison was performed via a one-way analysis of variance followed by Duncanˈs multiple range test (DMRT). P- Values of less than 0.05 (P<0.05) were considered as significant.

## Results

### Growth rate of *Chlorella vulgaris* cultivated under copper stress conditions

**Fig. 1:**
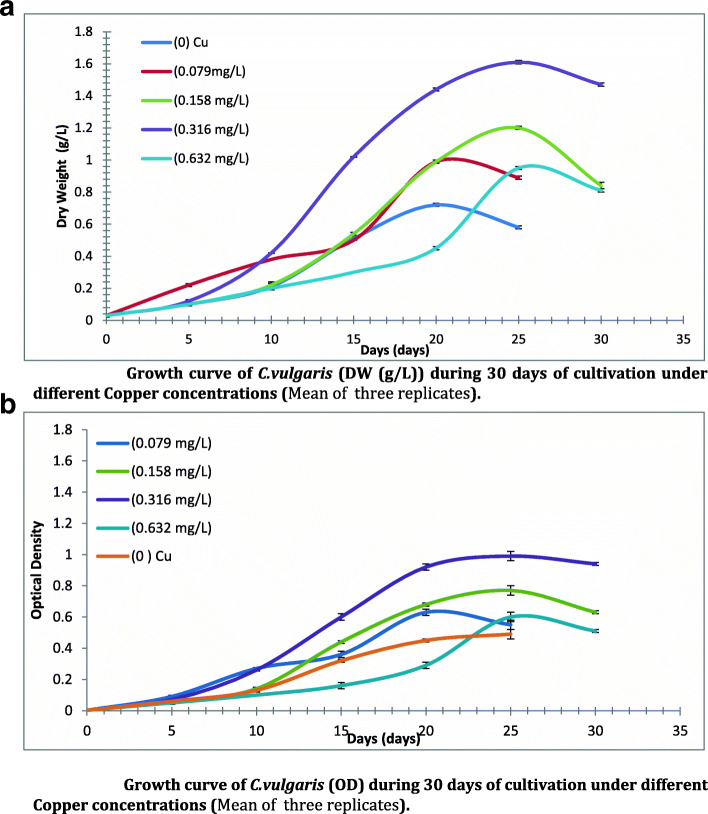
**a** Growth curve of *C.vulgaris* (DW (g/L)) during 30 days of cultivation under different Copper concentrations (Mean of three replicates). **b** Growth curve of *C.vulgaris* (OD) during 30 days of cultivation under different Copper concentrations (Mean of three replicates)

Growth rate of C.vulgaris (OD and DW) under the influence of Cu concentrations was recorded. At zero Cu (Cu depletion conditions) the alga grew well with a significant increase in growth parameters during the cultivation period, reached maximum at 25th day, except at day 30 where growth curve declined as shown in Fig. 1 (a and b). Also, the obtained data revealed that relative decrease in growth at high Cu concentrations (0.632 mg/L) compared to those recorded at 0.158 and 0.316 mg/L Cu especially towards the end of cultivation period (30 days) with 0.809–0.96% decrease from the growth values at 0.158 mg/L Cu and of 0.54–0.55% decrease from the growth values at 0.316 mg/L Cu.

### Antioxidant activity of promising fractions of *C.vulgaris*

Tables 1 and 2 tabulated the antioxidant activity of four promising fractions using ABTS and DPPH assays as a percentage of activity compared to both synthetic antioxidant (BHT) and natural standard (vitamin C), by determination of IC_50_ as μg/ml for each fraction, fractions [6, 7] had the lowest IC_50_ (highest antioxidant activity) recorded 11.65 and 12.93 μg/ml respectively against DPPH radical assay and 14.2 and 17.75 against ABTS radical assay compared to both BHT (11.2, 15.1) and vitamin C (12.9, 14.7 μg/ml) against DPPH and ABTS assays respectively.

**Table 1:** Antioxidant activity (as % and IC_50_) of the four promising algal fractions from the crude extract from {(0.316 mg/L (Cu) of *C.vulgaris* compared to BHT and vitamin C as standards against ABTS (%) radical method

Fraction	Hexane (100%)	Hexane:Chloroform (6:4)	Hexane:Chloroform (5:5)	Hexane:Chloroform (4:6)	BHT	Vit.C
Conc. (μg/ml)						
**500**	94.013±1.00^a^	96.220±1.070^a^	97.203±1.06^a^	94.253±1.092^a^	94.3±3.0^a^	97.1±3.6^a^
**250**	93.110±1.01^a^	95.237±1.081^a^	96.220±1.07^a^	89.013±1.000^b^	90.8±2.9^b^	93.2±2.5^b^
**125**	80.000±1.00^b^	85.157±1.036^b^	92.283±1.11^b^	85.110±1.018^c^	85.4±2.0^c^	90.2±2.1^c^
**62.50**	54.947±0.88^c^	68.170±0.662^c^	86.203±0.621^c^	71.073±0.793^d^	70.7±1.0^d^	87.9±1.9^d^
**31.25**	51.077±0.88^d^	56.000±1.000^d^	80.410±0.524^d^	66.190±0.734^e^	57.5±0.9^e^	76.9±1.9^e^
**15.62**	39.333±0.57^e^	40.610±0.385^e^	53.000±1.00^e^	44.600±0.529^f^	51.8±0.9^f^	53.0±1.0^f^
**IC**_**50**_	19.85	19.52	14.2	17.75	15.1	14.7

Different small letters on the column for each fraction indicate significant difference (*p*< 0.05). Error bars represent ±SD of three replicates

**Table 2:** Antioxidant activity (as % and IC_50_) of the four promising algal fractions from the crude extract {(0.316 mg/L (Cu) of *C.vulgaris* compared to BHT and vitamin C as standards against DPPH (%) radical method

Fraction	100% Hexane		Hexane:Chloroform (6:4)		Hexane:Chloroform (5:5)		Hexane:Chloroform (4:6)		BHT		Vit.C	
Conc. μg/ml. (μg/ml)												
	30 min.	60 min.	30 min.	60 min.	30 min.	60 min.	30 min.	60 min.	30 min.	60 min.	30 min.	60 min.
**500**	95.1±12.0^a^	96.1±1.6^a^	94.0±2.0^a^	95.1±2.1^a^	97.2±2.1^a^	97.1±2.1^a^	93.7±2.0^a^	92.±1.9^a^	96.3±1.5^a^	97.4±3.0^a^	93.4±1.1^a^	94.5±2.8^a^
**250**	82±1.34^b^	80.5±1.8^b^	83.3±1.9^b^	82.6±1.9^b^	86±1.20^b^	85.7±1.9^b^	80±1.4^b^	81.±1.8^b^	92.7±1.0^b^	93±2.2^b^	90.3±1.0^b^	90.9±2.2^b^
**125**	77±1.08^c^	77.5±1.6^c^	74.5±1.7^c^	75.8±1.3^c^	84.0±81.5^b^	83.9±1.4^c^	78.5±2.0^c^	77.1±1.3^c^	90.3±0.9^c^	89.0±2.0^c^	88.8±0.9^c^	88.3±1.9^c^
**62.50**	60.7±1.11^d^	61.1±1.7^d^	63.3±1.6^d^	64.8±1.3^d^	79.5±1.02^c^	80.7±1.9^d^	71±1.0^d^	70.9±1.9^d^	84.4±0.4^d^	84.9±2^d^	83.7±1.1^d^	85.9±1.0^d^
**31.25**	59.3±0.9^d^	60.9±0.9^d^	56.3±1.1^e^	55.1±1.1^e^	74.4±1.2^d^	75.9±1.4^e^	60.3±0.9^e^	61.9±1.2^e^	72.7±0.5^e^	73.2±1.9^e^	68.2±0.7^e^	69.5±0.9^e^
**15.62**	57.5±0.8^e^	56.9±0.7^e^	53.5±1.2^f^	54.0±0.9^e^	65.8±1.3^e^	67.09±1.^f^	59.3±0.7^e^	60.4±1.1^e^	70.2±0.8^f^	69.4±1.2^f^	64.4±0.5^f^	63.0±0.9^f^
**IC**_**50**_	13.58	13.72	14.59	14.46	11.87	11.65	13.18	12.93	11.1	11.2	12.1	12.9

Different small letters on the column for each fraction indicate significant difference (*p*< 0.05). Error bars represent ±SD of three replicates

### Antioxidant and anticancer activities of the crude extract and fractions

**Fig. 2:**
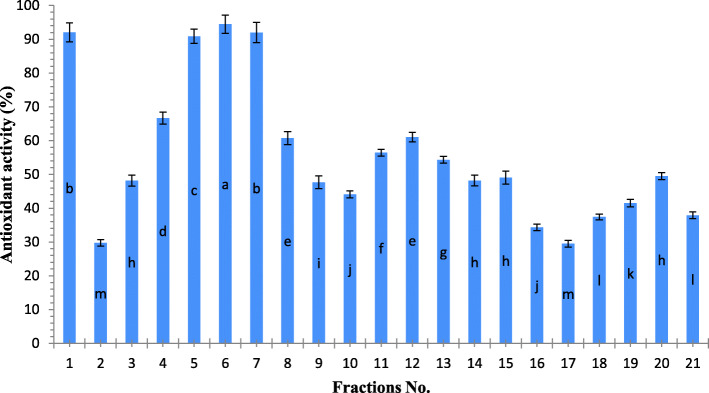
Antioxidant activity (as %) of 21 fractions at 200 μg/ml of the *C. vulgaris* 0.316 mg/L (Cu), using ABTS radical method

Figure 2 showed the antioxidant activity (as %) of 21 fractions resulted from fractionation of the promising crude extract (0.316 mg/L Cu *C. vulgaris*) using ABTS assay. From the obtained results it was clear that, there were nine fractions gave high activity (more than 50%) as the followings: (1, 4–8, 11–13, and). Whereas, fractions 1, 6, 7 showed significantly (*p*< 0.05) the highest activity (92.05±2.02%, 94.43±1.78% and 92.00±1.65% respectively).

**Fig. 3:**
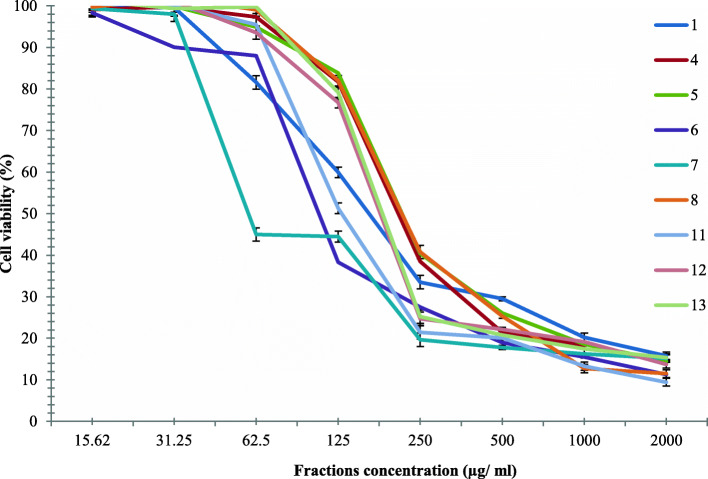
Anticancer activity of the nine promising fractions from 0.316 mg/L (Cu) of *C.vulgaris* against cervical cell line (Hela cell line) as % of cell viability (Mean of three replicates)

The anticancer activity of nine fractions (1, 4–8, 11–13, and) which recorded antioxidant activity more than 50% using ABTS radical scavenging method were examined against Hela cell line as a percentage of % of cell viability as shown in Fig. 3.

A decline in IC_50_ (higher anticancer activity) of fractions compared to their crude extract (IC_50_ = 90.2 μg/ml) may be due to synergistic effects between bioactive compounds of fractions especially fraction [6, 7]. The promising active ingredients of the promising fractions were identified using GC-MS, as shown in Table 3.

**Table 3:** GC/mass for most promising *C. vulgaris* fractions cultivated under Cu (0.316 mg/L) as relative percentage

No.	Compound Name	M.wt	(Fractions number)				Biological activity	References
			**1**	**5**	**6**	**7**		
1	4 h-1-benzopyran-4-one, 2-(3,4-dimethoxyphenyl)-3,5- dihydroxy-7-methoxy-	344				5.87	Antioxidant, antimicrobial, cancer enzyme inhibitors in pharmaceutical, cosmetics, and food industries	[45]
2	3,7,11,15-Tetramethyl-2-hexadecen-1-ol	296	2.95	1.33		2.52	anticancer, anti-inflammatory and antimicrobial,antioxidant activities	[46]
3	1-Nonadecene	266	1.10	3.14	6.02		Antimicrobial, antioxidant activity	[47, 48]
4	hexadecanoic acid, methyl ester	270	21.28	11.77	3.87	5.30	Antioxidant, antimicrobial,, hemolytic, hemolytic, 5-alpha reductase inhibitor cancer enzyme inhibitors in pharmaceutical, cosmetics, and food industries	[45, 49]
5	9-octadecenoic acid (z)-, methyl ester	296	23.02	57.86	6.39	9.05	Antioxidant, antimicrobial, cancer enzyme inhibitors in pharmaceutical, cosmetics, and food industries	[45, 49, 50]
6	1,2-benzenedicarboxylic acid	390	3.16		27.4	46.89	Antioxidant,antifouling, antimicrobial, cancer enzyme inhibitors in pharmaceutical, cosmetics, and food industries	[45, 49]
7	pentacosane	352	9.97	3.48			Antioxidant and antimicrobial activity	[51]
8	dotriacontane	450	2.11	2.83		1.38	Antimicrobial, antifungal anti-inflammatory, cytotoxic activity	[47]
9	phthalic acid, butyl tetradecyl ester	418		7.41		3.91	Antimicrobial activity	[46]
10	4 h-1-benzopyran-4-one,2-(3,4dihydroxyphenyl)-6,8-di-á-d-glucopyranosyl-5,7-dihydroxy-	610			1.17	3.33	Antioxidant, antimicrobial, cancer enzyme inhibitors in pharmaceutical, cosmetics, and food industries	[45]
11	7,9-di-tert-butyl-1-oxaspiro [4.5]deca-6,9-diene-2,8-dione	276		1.60	1.36		Antimicrobial activity	[52]
12	1-hexadecanol, 2-methyl-	256		1.71			anticancer, anti-inflammatory and antimicrobial,antioxidant activities	[49]
13	6,9,12,15-docosatetraenoic acid,methyl ester	346			1.79	4.18	phytopharmaceutical importance	[53]
14	Neophytadiene	278	5.43	4.27	4.06		Antioxidant, antibacterial activity	[54]
15	Methyl 4,7,10,13-hexadecatetraenoate	262	3.61				Antioxidant, antibacterial activity	[54]
16	Phytol	296	12.08				Antioxidant, antibacterial activity	[54]
17	9,12,15-octadecatrienoic acid,methyl ester, (z,z,z)-	292	14.49				Antimicrobial, antioxidant, anticancer	[45]
18	heptanoic acid, docosyl ester	438	0.93				Antioxidant and antifungal activity	[55]
19	3,3,3-Trifluoro-1-piperidin-1-yl-2-trif luoromethyl-propan-1-one	263		1.49				
20	1,4-benzenediol, 2-(1,1-dimethylethyl)-5-(2-propenyl)-	206		3.10			Anticancer, Antioxidant activity and pesticidies	[56]
21	1,3-Dioxolane, 2-heptyl-4-phenyl-	248			4.32		Anti-inflammatory, anticarcinogenic and ant aging	[57]
22	Cycloheptasiloxane,tetradecamethyl-	518			1.34		antibacterial, antifungal, antifouling, immunomodulatory and antitumor activities	[58]
23	Corymbolone	208			2.04		Antioxidant, antimicrobial activity	[59]
24	spiro[4.5]decan-7-one, 1,8-dimethyl-8,9-epoxy-4-isopropyl-	236			1.88		Anticancer activity	[60]
25	8-(2-Acetyloxiran-2-yl)-6,6-dimethyl octa-3,4-dien-2-one	236			3.28			
26	03027205002 flavone 4′-oh,5-oh,7-di-o-glucoside	428			1.21		Antioxidant activity	[61]
27	1,1'-bicyclopropyl]-2-octanoic acid, 2'-hexyl-, methyl ester	236			1.88		Anticancer activity	[53]
28	2-acetyl-3-(2-cinnamido)eth yl-7-methoxyindole	362			2.03	1.31	Antioxidant and antibacterial activity	[62]
29	1,2-benzenedicarboxylic acid, bis(8-methylnonyl) ester	446			3.35		Antioxidant, antimicrobial, anticancer	[45]
30	13-docosenamide, (z)-	337			24.85		Antioxidant, antitumor activity	[63]
31	2,2-dideutero octadecanal	270				2.01	Antimicrobial activity	[47]
32	hexadecanoic acid, 3[(trimethylsilyl)oxy] propyl ester	386				3.65		
33	Hexanoic acid, 2-ethyl-,oxybis(2,1-ethanediyloxy-2,1-ethane diyl) ester	446				2.11		
34	9,10-secocholesta-5,7,10(19)-triene-1,3-diol	488				1.05	Antiviral activity	[46]
35	S-(1,3-diphenylbutyl) dimethylthiocarbamate	311				2.06	Antioxidant and anticancer activity	[64]
36	Astaxanthin	596				2.18	Antioxidant activity	[65]

### Separation, characterization and identification of isolated bioactive compound

**Fig. 4:**
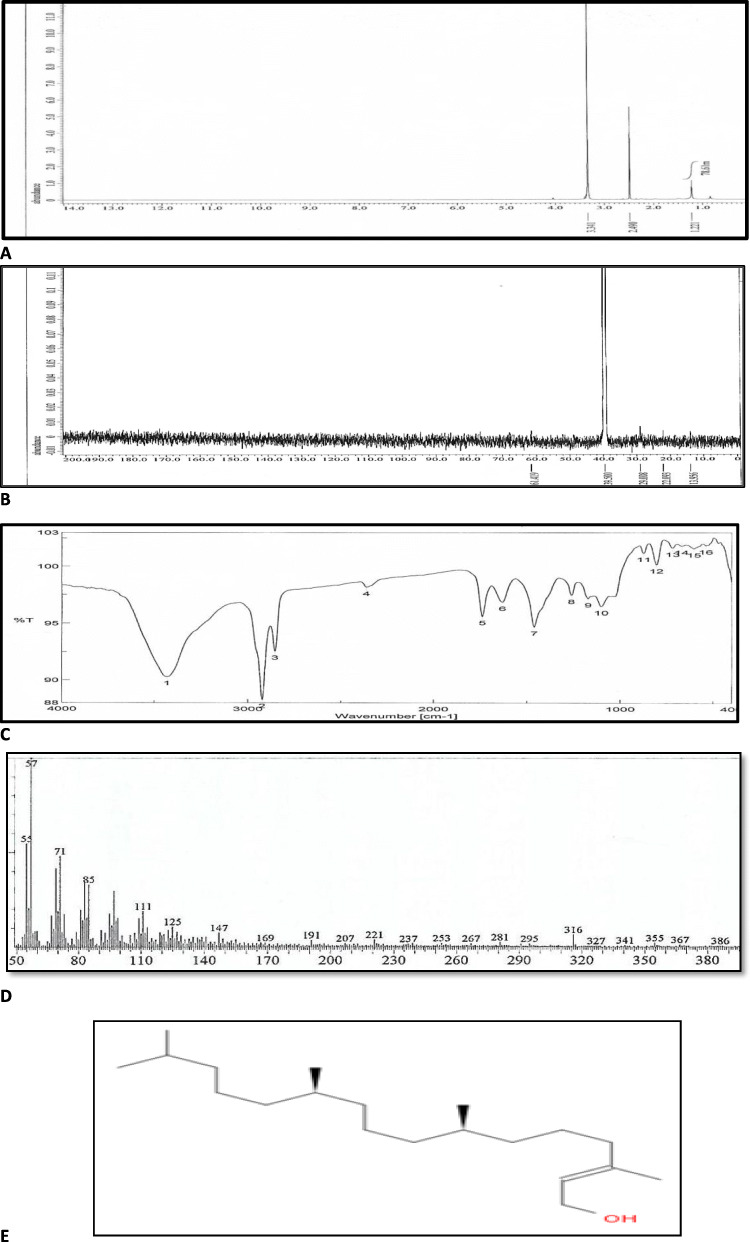
H1-NMR spectrum (**a**); C13-NMR (**b**); IR-Spectrum (**c**); Mass spectrum (**d**); The chemical structure (**e**) of separated compound from *Chlorella vulgaris*

Twenty-one Fractions were separated from *Chlorella vulgaris* (grown under 0.316 mg/L copper ion) crude extract (Fig. 2). Fraction No. 7 showed the highest biological activities as antioxidant and anticancer (Figs. 2 and 3). For separation of active compound(s) from fraction number 7, a Precoated TLC F254 and Ethyl acetate: Hexane (9:1) as mobile phase where be used. Additionally, two-dimension TLC was performed using different solvents combinations (9.5: 0.5, 9:1, 8.5:1.5 ethyl acetate: hexane respectively). The obtained pure compounds with R_f_ 0.56 and color ranged from yellow to orange (with maximum absorption at 445 nm) was identified by different spectroscopic analyses. The obtained data of the spectroscopic analyses and colorimetric maximum absorption revealed that terpenoids derivative was identified with the common fragment ions: 57, 71, 85, 149, 169, 221 and 267 Da and the molecular ion of m/e= 296 is consistent with the molecular formula of C_20_H_40_O (Fig. 4d).

These results were confirmed by H^1^-NMR and C^13^-NMR. The H^1^-NMR data (Fig. 4 A and B) which indicated that the pure compound had the following type of protons; A signal at δ 3.342 μg/ml was characteristic of protons of O-CH_2_ group; the singlet signal at δ 2.490 μg/ml was characteristic of the protons of methylene (CH_2_) group and the singlet signal at δ 1.221 μg/ml was characteristic of protons of methyl (CH_3_) group. in addition, The C^13^-NMR data (Fig. 4b). indicated that the pure compound had the following type of carbons; A signal at δ 61.419 μg/ml was characteristic of O-CH_2_-R group and the signal at δ 39.500 μg/ml was characteristic of R-CH_2_- electronegative group. in addition, A signal at δ 29.008 and 22.093 μg/ml was characteristic of R-CH_2_-R group, However, A signal at δ 13.956 μg/ml was characteristic of R-CH3 group. Also, FTIR was used to analyze the functional groups in the separated compound. In the FT-IR spectral analysis of pure compound from *Chlorella vulgaris* observed characteristic bands corresponding to O-H stretching vibration at 3431.71 cm^− 1^. The C-H stretching was observed at 2923.56 and 2855.1. The absorption peaks at 1630 was assigned to C=C group (Fig. 4c). The obtained data indicated that the separated compound was exactly matched with the reported data for (2E,7R,11R)-3,7,11,15-Tetramethyl-2-hexadecenol (Fig. 4e). The MS spectrum of separated compound from *C.vulgaris* identified the common fragmental ions as: 57, 71, 85, 149, 169,221 and 267 Da with molecular ion m/e=296 Da (as shown in Fig. 4d. Qualitative test was carried out on the pure compound showed a positive result for terpene (reddish brown color).

### Biological activities of isolated compound

#### Antioxidant activity

Table 4, recorded the antioxidant activity of pure compound compared to those synthetic antioxidant (BHT), and natural antioxidant (Vit. C), using DPPH radical assay, the results showed the higher antioxidant activity of pure compound with lower IC_50_ (10.59 μg/ml), compared to BHT (11.2 μg/ml), and Vit. C, (12.9 μg/ ml) after 60 min of incubation.

**Table 4:** Antioxidant activity as (% and IC_50_) of isolated compound from *C.vulgaris* (0.316 mg/L Cu) compared with (BHT) and (vitamin C) against DPPH (%) radical method

Compound	Isolated compound		BHT		Vitamin C	
conc. μg/ml (μg/ml)						
	**30 min.**	**60 min.**	**30 min.**	**60 min.**	**30 min.**	**60 min.**
**250**	84.1±1.02^a^	85.32±1.22^a^	92.7±1.0^a^	93.2±2.2^a^	90.3±1.0^a^	90.9±2.2^a^
**125**	76.99±1.1^b^	80.24±1.35^b^	90.3±0.9^b^	89.0±2.0^b^	88.8±0.9^b^	88.3±1.9^b^
**62.50**	72.74±1.2^c^	77.04±1.24^c^	84.4±0.4^c^	84.9±2.0^c^	83.7±1.1^c^	85.9±1.0^c^
**31.25**	69.6±1.9^d^	73.74±1.02^d^	72.7±0.5^d^	73.2±1.9^d^	68.2±0.7^d^	69.5±0.9^d^
**15.62**	68±0.97^d^	73.74±1.00^d^	70.2±0.8^d^	69.4±1.2^e^	64.4±0.5^e^	63.0±0.9^e^
**IC**_**50**_	11.48	10.59	11.1	11.2	12.1	12.9

Different small letters on the column for each time indicate significant difference (*p*< 0.05) Error bars represent ±SD of three replicates

#### Anticancer activity and prooxidant effect

**Fig. 5:**
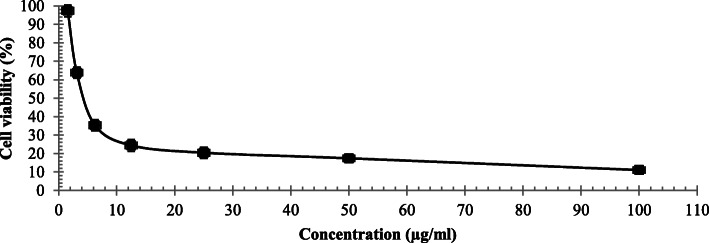
cell viability (%) of pure compound against Hel cell line with IC_50_ = 4.38 μg/ml) (Mean of three replicates)

Cytotoxicity of pure compound against Hela cell line recorded in Fig. 5 showed that, the viability of cancer cells mostly was affected by the lowest extract concentration (100–0.1 μg/ml) with IC_50_ (4.38 μg/ml) compared to DOX as synthetic drug (13.3 μg/ml).

## Discussion

This study revealed declined growth of *C.vulgaris* at high copper concentration (0.632 mg/L). It is worth noting that crude extract, fractions and pure compound from Chlorella sp. (grown under 0.316 mg/L copper ion) were investigated and the results revealed high potential for use of *Chlorella vulgaris* as antioxidant and anticancer sources against DPPH and Hela cell lines respectively.

The stimulatory effect of Cu at low concentration in this study may be due to its involvement in different metabolic processes or in the production of certain organic compounds that induce decreased Cu toxicity. These results coincided with those of both [45, 46], they suggested that some algae may synthesize metal binding compounds to sequester copper ions in the cytoplasm and reduced its toxicity as in *Euglena gracilis* and *Thalassiosira pseudonana*. However, the declined growth of *C.vulgaris* at high copper concentration may be due to the decreased resistance of *C.vulgaris* to tested Cu concentrations (from 0.158 mg/L to 0.632 mg/L). Also, copper, seems to regulate the expression of functional activity as concentration factor for different algal enzymes as reported by [47]. These results were in agreement with results obtained by [48] who reported that, copper is acutely toxic to *Padina boergesenii* at concentrations higher than 500 mg Cu/L. Moreover, low concentration of copper is toxic to *P. boergesenii* after a prolonged exposure of 21 days. Both growth and photosynthesis of *Chlorella vulgaris* were dependent on both concentration and exposure time under the influence of sub-lethal concentrations of the heavy metals Cu, Cr, Zn, Cd and Pb [49].

The antioxidant activity of the promising extracts and fractions may be correlated to the presence of different active groups such as hydroxyl group and unsaturated bonds in the chemical structure of its compounds which showed high ability for scavenging free radicals and prevent the oxidation processes (as shown in Table 3), from the data of Table 3 We can conclude that the promising extract are rich with various bioactive compounds as antioxidant such as 4 h-1-benzopyran-4-one,2-(3,4-dimethoxyphenyl)-3,5-dihydroxy-7-methoxy, 3,7,11,15-Tetramethyl-2-hexadecen-1-ol, hexadecenoic acid, methyl ester, 9-octadecenoic acid (z)-, methyl ester, 1,2-benzenedicarboxylic acid, Neophytadiene, 9,12,15-octadecatrienoic acid, methyl ester, (z,z,z)-, Astaxanthin. These observations were in agreement with the previously published studies [50–63].

Fractions (1, 5–7, and) exhibited the highest anticancer activity with the lowest IC_50_ values (162.30, 154.7, 84.2, 40.0 μg/ml respectively) as there is a strong correlation between anticancer activity and their richness contents of anticancer compounds as mentioned in Table 3 such as (4 h-1-benzopyran-4-one, 2-(3, 4-dimethoxyphenyl)- 3, 5- dihydroxy-7-methoxy, 3,7,11,15-Tetramethyl-2-hexadecen-1-ol, hexadecanoic acid, methyl ester, 9-octadecenoic acid (z)-, methyl ester, 1,2-benzenedicarboxylic acid, dotriacontane, 1-hexadecanol, 2-methyl, 1,3-Dioxolane, 2-heptyl-4-phenyl, Cycloheptasiloxane, tetradecamethyl) as reported by many studies [64–71]. Regarding to cancer and synergy studies, Eichhornia sp. compounds have shown synergistic effect between them, especially on Hela cancer cell models [2]. Mohd Syahril [72] suggests that new anticancer natural products of chloroform extract from unicellular green algae (Chlorella sp) and filamentous microalgae (Spirulina sp) are possible, Recently, the anticancer properties of some algae-derived resources have been found to modulate several cellular mechanisms such as cellular cytotoxicity, downregulate invasion of tumor cells, and enhancement of cancer cells apoptosis [73] C-Phycocyanin from microalgae *Spirulina platensis* has the ability to induce pathologic alteration and DNA fragmentation, upregulates Fas and ICAM expression, downregulates expression of Bcl-2, as well as activation of caspases 2,3,4,6,8,9,10 in HeLa cell line [74].

From the previous antioxidant and anticancer activities, Fraction 1, 5, 6 and 7 revealed the highest activity throughout. It was obvious that the antioxidant activity of fractions was higher than that showed by their original crude extract (13.4, 20.5 μg/ml against DPPH and ABTS respectively), it may be due to the synergistic effect between bioactive compounds that found in the fractions as recorded in Table 3 and antagonistic effect in crude extract.

Our results were parallel to those obtained by [75] They reported that apoptosis was 2.5-fold higher in the case of the Chlorella sp. ethanol extract against human colon carcinoma cell line (HCT116) when compared with other microalgae strains. Moreover, [76] studied the interaction effect of herbal extract and ascorbic acid on antioxidant activity suggesting that antagonistic interaction between combination of either ascorbic acid and herbal extracts should be taken in consideration to prevent any health potential complications.

The isolated pure compound was recently extracted and identified from *Chlorella vulgaris* as reported by [77]. The antioxidant activity of this pure compound may be due to the presence of hydroxyl group and double bond which help for scavenging and react with radical species (ROS and RNS) as reported by Shanab et al. [53].

**Fig. 6:**
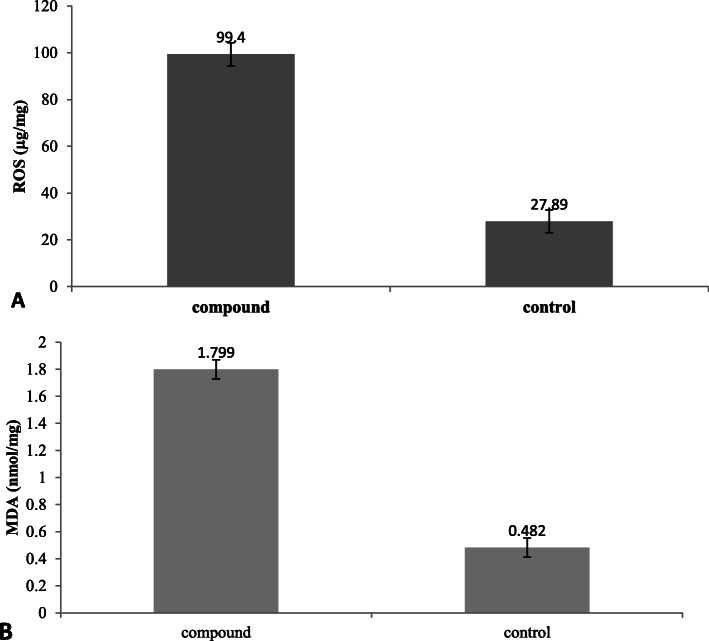
Effect of pure compound on A): ROS; B): MDA production of Hela cell line

Also, these results were in agreement with the results obtained by Mohamed [51] who proved the antioxidant and anticancer activity of the crude extract continuing the pure bioactive compound (as indicated in GC/MS analyses). in addition to the results obtained by [78] who proved that, the methanolic extract of *Kirganelia reticulata* exhibited anticancer activity due to bioactive compounds separated from GC-MS analyses that include the pure compound ((2E,7R,11R)-3,7,11,15-Tetramethyl-2-hexadecenol). Additionally, the obtained results may be due to the prooxidant effect of isolated compound which led to increase the production of ROS and MDA of Hela cell line as shown in Fig. 6 A and B). These results were in agreement with results obtained by [79] who found that, some antioxidants act as prooxidants inducing nuclear damage and lipid peroxidation in presence of transition metal. Also, Seo and Lee [80] revealed that, some antioxidants have both prooxidant and antioxidant it can inhibit the proliferation of cancer cells. They also have shown antiproliferative effects on human colon, breast, prostate, and oral cancers. In addition to, some antioxidant compounds can act as pro-oxidant agent and increase the ROS production in cancer cell [81–84]. However, [83] revealed that those compounds are antioxidants in lower concentration but can be a pro-oxidant at a high level.

Accumulation of ROS disturbs the redox control of cell cycle progression through phosphorylation and ubiquitination of cell cycle proteins, resulting in aberrant cell proliferation and apoptosis [85]. Though ROS is dangerous to cells, the anticancer role of numerous treatments depends on their ability to encourage controlled ROS production, that changes cellular redox balance leading to oxidative stress, damage to mitochondria, and finally apoptosis induction [86], mitochondrial destabilization, caused by the increased ROS production, which in turn regulated the expression of apoptotic proteins. Pro-oxidant effect induced by polyphenols (as polyhydroxy compounds) led to generate ROS and produce [87] cell cycle arrest, and [88] induction of apoptosis and DNA fragmentation in cancer cells.

**Fig. 7:**
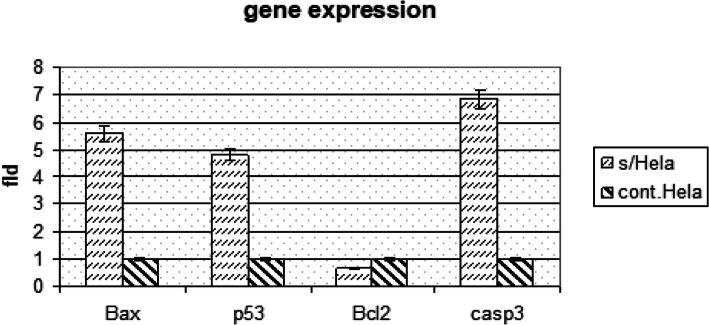
Evaluation of pro-apoptotic and anti-apoptotic genes expression in Hela cell post treatment with pure compound for 48 h using real-time PCR compared to the non-treated cell control (Mean of three replicates.)

The anticancer potential of pure compound on Hela cells was proved through evaluating the expression of pro-apoptotic and anti-apoptotic genes. Data recorded in Fig. 7 revealed that pro-apoptotic genes p^53^, caspase 3 and Bax were significantly up-regulated/ over expressed after treatment with wheatgrass for 48 h compared to cell control values. Also, anti-apoptotic genes Fig. 7 showed that, the untreated control cells showed a normal spindle intact cells that wasn’t clear in treated cells, where cells were detached showed ruptured membrane and irregular cell membrane. Bcl-2 was significantly down-regulated compared to cell control values.

Apoptosis is a gene-regulated cell death mechanism with well-described biological changes [89]**.** A lot of bioactive compounds from algae can inhibit cancer cell growth through the induction of apoptosis; so, elucidating the mechanism of apoptosis has significant implication in cancer chemoprevention. Increased Bax expression can induce apoptosis and increased Bcl-2 expression which may inhibit apoptosis [90]. Bcl-2 proteins prevent apoptosis by decreasing the cytochrome C release to inhibit activation of caspase 3. However, Bax protein, helps in the transfer of cytochrome C across the membranes thus forming apoptotic bodies and activates both caspase 9 and caspase 3 that finally leads to apoptosis [91].

Increased ROS in cancer cells is also accompanying with the activation of a key signaling protein p^53^. Also, p^53^ accumulates in the nucleus and controls the pro apoptotic members expression, Bax and PUMA. Once up regulation of PUMA occurs, PUMA that binds to Bcl-2, releasing p^53^ to activate Bax [92].

On the other hand, Fusi [93] reported the antioxidant compounds increases sirtuin 1 (SIRT1) expression and monophosphate activated protein kinase (MAPK) activation in Hela cell line. Safflower polysaccharide represents a major active component of *Carthamus tinctorius*. It inhibited proliferation and also increased apoptosis of Hela cells by down regulation of the phosphatidylinositol-3-kinase/ AKT pathway [94]. Additionally, *Terminalia sericea* enabled caspases-7 and -8 and poly (ADP-ribose) polymerase (PARP) in HeLa cancer cell line [83]. Also, pyrogallol induces superoxide anion, and this generates activation of caspase-3 and phosphatidylserine [95].

Moreover, [96] evaluated the induction of apoptosis by isolated bioactive compounds (1-(2-hydroxyphenyl)-4-methylpentan-1-one (C1) and 2-[(3-methylbutoxy) carbonyl] benzoic acid (C2)) from Rubus fairholmianus against MCF-7 breast cancer cells. Reactive oxygen species (ROS) production after treatment with C1 and C2 was found to be higher inducing nuclear damage. Expression of apoptotic proteins (caspase 9, p^53^, and Bax) after the treatments was significantly up regulated as indicated using immunofluorescence.

## Conclusion

The present study verifies the idea that oxidative stresses especially copper stress condition led to increase the biological activities of *Chlorella vulgaris* as antioxidant and anticancer, and these activities related to active ingredients increased in organic extract of algal species cultivated under stress condition and this due to alteration in gene expression of algal cells, the chromatographic and spectroscopic results proved the chemical structure of pure compounds as (2E,7R,11R)-3,7,11,15-Tetramethyl-2-hexadecenol, and this compounds has high antioxidant and anticancer activity when compared with synthetic and natural standards. The author recommends cultivation of *Chlorella vulgaris* in large scale under various stress conditions for use the crude extracts and semi purified fractions for making a pharmaco-economic value in Egypt and other countries.

## Data Availability

The data used and analysed in this study are available from the corresponding author on reasonable request.

## Abbreviations

ABTS: 2, 2′- azino-bis ethylbenzthiazoline-6-sulfonic acid
DMSO: Dimethyl sulfoxide
BHT: Butylated hydroxy toluene
DPPH: 2,2-diphenyl-1-picrylhydrazyl
DOX: Doxorubicin
GC-MS: Gas chromatography-Mass spectrum
MAPK: Monophosphate activated protein kinase
MDA: Malondialdehyde
MTT: 3-(4,5-dimethylthiazol-2-yl)-2,5-diphenyltetrazolium bromide
ROS: Reactive oxygen species
RT-PCR: Real-time Polymerase Chain Reaction
TLC: Thin layer chromatography
